# Incontinentia Pigmenti: A Rare Case of Survival of a Male Infant

**DOI:** 10.7759/cureus.80063

**Published:** 2025-03-04

**Authors:** Riley Shin, Helen Chen, Michelle Tarbox

**Affiliations:** 1 School of Medicine, Texas Tech University Health Sciences Center, Lubbock, USA; 2 Dermatology, Texas Tech University Health Sciences Center, Lubbock, USA

**Keywords:** blaschkoid distribution, incontinentia pigmenti, inheritance pattern, linear hyperpigmentation, somatic mosaicism

## Abstract

This case describes a seven-month-old male infant with incontinentia pigmenti (IP), a rare X-linked dominant disorder that is typically lethal in male fetuses. The infant presented with blaschkoid hyperpigmentation but showed no neurologic, dental, or ocular abnormalities. Genetic testing revealed a normal karyotype without *IKBKG *(inhibitor of nuclear factor kappa B kinase regulatory subunit gamma) mutations, suggesting possible somatic mosaicism, a rare survival mechanism in male patients with IP. This case underscores the rarity of IP in male infants and highlights the need for multidisciplinary follow-up to monitor potential extracutaneous involvement. These findings contribute to the limited understanding of male presentations of IP and their clinical management.

## Introduction

Incontinentia pigmenti (IP), also known as Bloch-Sulzberger syndrome, is a rare genetic disorder caused by a mutation in the *IKBKG* (inhibitor of nuclear factor kappa B kinase regulatory subunit gamma) gene. This gene encodes NEMO (NF-kappa-B essential modulator), a regulatory protein in the NF-κB (nuclear factor kappa B) signaling pathway necessary for proper embryogenesis. Approximately 80% of IP cases result from deletions of exons 4-10 in the *IKBKG* gene located on chromosome Xq28. This X-linked dominant mutation occurs in approximately 1 in 50,000 newborns and is typically lethal in male fetuses, with a female predominance of 37:1 [1]. IP is characterized by distinctive cutaneous manifestations, along with abnormalities in the teeth, eyes, hair, and central nervous system (CNS). This condition occurs in less than 3% of the male population, with survival attributed to one of three potential mechanisms that prevent embryonic lethality: somatic mosaicism, where a post-zygotic mutation allows partial NF-κB expression in some cells, Klinefelter’s syndrome, which provides an extra X chromosome that may carry a functional gene copy, or hypomorphic mutations, which cause a milder NF-κB impairment [2].

## Case presentation

A seven-month-old male infant presented with small, red, blistering lesions that had been present since birth. These lesions progressed to warty plaques and eventually healed into stripe-like hyperpigmented lines along the trunk and extremities, with mild involvement of the face and ears. The mother denied any family history of similar rashes. The infant exhibited no signs of neurological issues or a history of seizures and had met all developmental milestones to date. Dental abnormalities were not assessed, as he had not yet developed teeth.

**Figure 1:**
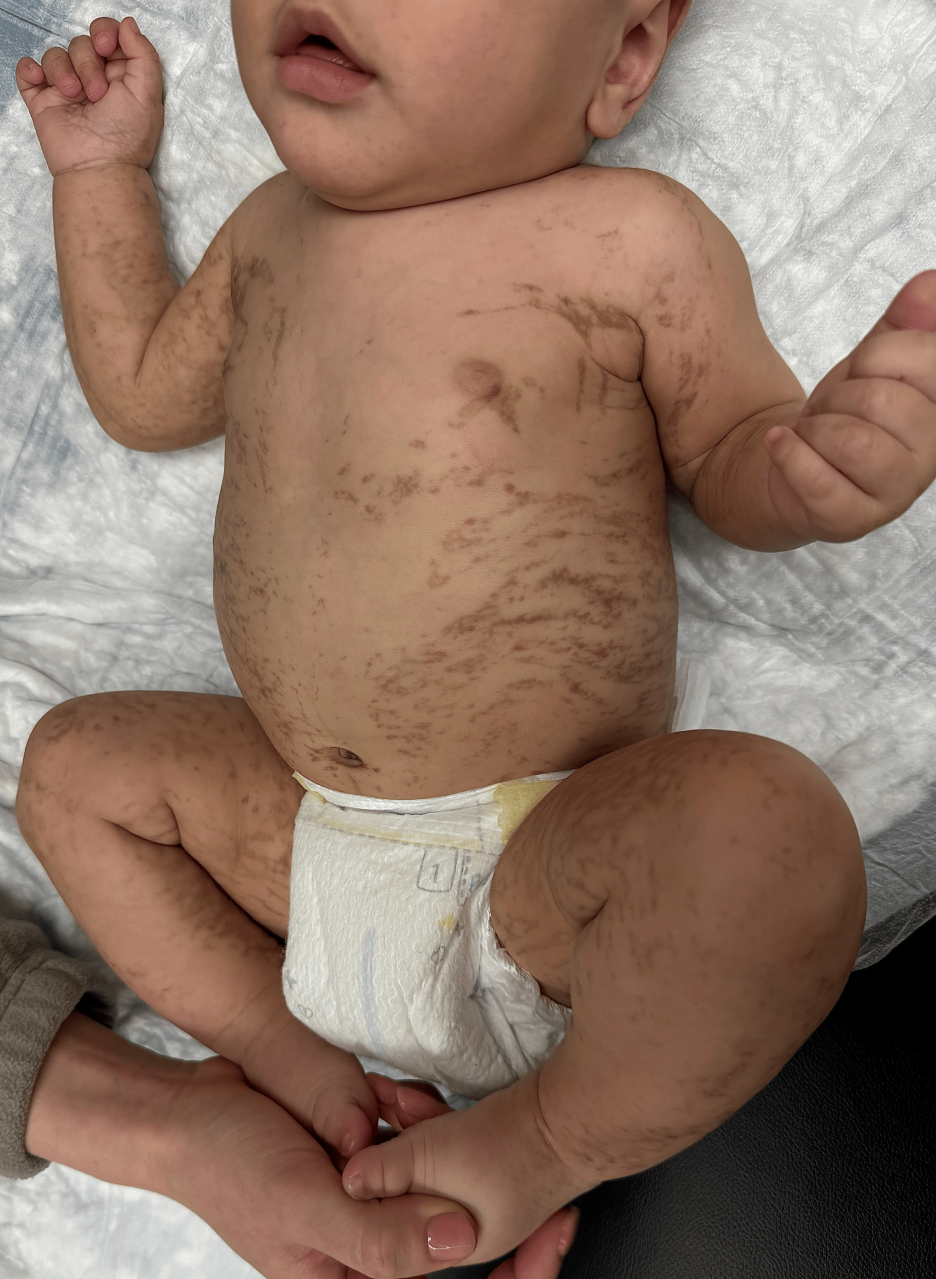
Stage three of incontinentia pigmenti characterized by a blaschkoid distribution of hyperpigmented streaks and whorls on the bilateral ears, neck, trunk, upper extremities, and lower extremities

**Figure 2:**
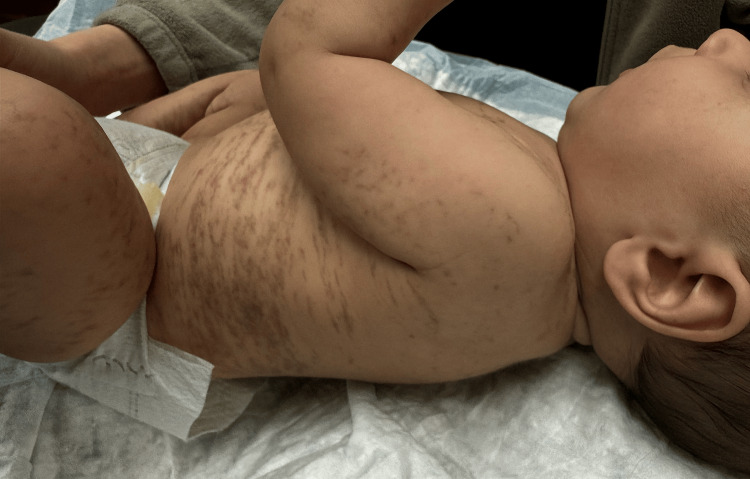
Hyperpigmented linear to reticulated streaks following Blaschko’s lines, observed on the left side of the infant, characteristic of the third stage of incontinentia pigmenti

On physical examination, hyperpigmented streaks and whorls were observed in a blaschkoid distribution on the bilateral ears, neck, trunk, upper extremities, and lower extremities suggestive of stage three IP (Figures 1, 2). The parents were counseled on the inheritance pattern and potential associations of IP, including developmental delays, seizures, and dental abnormalities. They were advised to continue regular well-child visits and seek a pediatric ophthalmology evaluation to rule out retinal vascular involvement.

At a four-month follow-up, an ophthalmologic evaluation found no retinal vascular involvement. Genetic testing revealed a normal karyotype with no *IKBKG* mutation, suggesting a somatic mosaicism variant. This aligns with previous findings, where 19 out of 28 reported cases of male patients with IP showed no detectable *IKBKG* mutation in blood, likely due to tissue-specific mosaicism [3]. However, despite the genetic findings, the clinical course of skin lesion development strongly supports IP. Given the potential morbidity associated with ocular and CNS involvement, close multidisciplinary follow-up is warranted.

## Discussion

The cutaneous presentation of IP progresses through four distinct stages: inflammatory vesicular (papules, vesicles, and eosinophilic pustules), verrucous (warty plaques), hyperpigmented (linear or whorled brown pigmented lesions), and hypopigmented (atrophic lesions), typically following Blaschko’s lines. Early vesiculopustular lesions resolve through keratinocyte apoptosis, allowing replacement by normal genotype cells and facilitating progression through these stages [4]. Consequently, the hyperpigmentation observed in our patient, indicative of stage 3 IP, is expected to gradually fade with age and transition into linear areas of hypopigmentation.

With no familial history of IP, diagnosis requires the presence of at least one major criterion, such as neonatal vesicular rash, hyperpigmentation, or linear atrophic lesions [5]. Our patient met two major criteria: a neonatal vesicular rash reported by the parent and current hyperpigmentation. No minor criteria have been observed yet, though they may develop over time. The prevalence of these minor criteria sequelae is as follows: dental (80%), hair (28-38%), nail (40%), ocular (25-77%), and neurological (33%) [1].

Genetic testing confirmed that our patient has a normal karyotype (46, XY), suggesting that survival is due to somatic mosaicism. Somatic mosaicism arises from a postzygotic mutation that partially impairs NF-κB function, allowing a subset of cells to retain normal function and sustain viability [2]. The clinical manifestations of IP in male patients are highly variable, likely reflecting the underlying mosaicism. For example, Gupta et al. described a 5-year-old male patient who developed vesicular lesions that progressed to hyperpigmentation, along with hypodontia, intellectual delay, and primary nocturnal enuresis [6]. A case reported by Kenwrick et al. involved a male patient with severe hypodontia, sparse hair, and vision loss due to spontaneous vitreous hemorrhage. In contrast, other cases exhibited no systemic involvement, with affected male patients presenting only with IP-consistent skin findings [7]. These cases demonstrate varying degrees of cutaneous and systemic involvement, reinforcing the broad clinical spectrum of IP in the male population.

The variability in disease severity in the male population with suspected somatic mosaicism raises the broader question of whether sex influences the overall disease burden in IP. Pacheco et al. found that in eight of nine male patients with a normal karyotype and no familial history of IP, lesions were confined to a single extremity, unlike female patients who exhibited a broader distribution along Blaschko’s lines [8]. However, this pattern was not observed in our patient, further underscoring the variability in cutaneous involvement. This difference may be attributed to the timing and extent of the postzygotic mutation leading to somatic mosaicism. While cutaneous patterns in males often differ from those in females, Minić et al. found no significant differences in the prevalence of CNS abnormalities between sexes in a study of 795 IP patients (719 female patients and 76 male patients), suggesting that other extracutaneous symptoms may follow similar trends [9]. Although genetic factors affect survival rates between sexes, males do not necessarily have more severe systemic involvement. Both sexes can develop complications with varying severity, emphasizing the need for further research to understand sex-based differences in the clinical presentation of IP. 

While somatic mosaicism is the most common survival mechanism in males, Klinefelter syndrome (47, XXY) also enables survival by creating a heterozygous genotype in which X-inactivation favors the wild-type *IKBKG* allele [2]. Additionally, hypomorphic mutations arising in early embryonic development can result in a less deleterious form of *IKBKG*, providing another segue for survival. However, whereas somatic mosaicism and Klinefelter syndrome do not significantly impact disease severity based on sex, hypomorphic mutations produce a distinct phenotype. Females typically present with milder symptoms, often asymptomatic or limited to cutaneous manifestations, while males deviate from the typical IP phenotype and exhibit hypohidrotic ectodermal dysplasia and severe immunodeficiency [7,10].

## Conclusions

This case demonstrates a male infant with IP, illustrating the rarity of survival of male infants and highlighting somatic mosaicism as a potential mechanism. While cutaneous progression is crucial for diagnosis, its severity does not necessarily predict systemic involvement. Given the risks of neurological and ophthalmological complications, multidisciplinary care and close long-term follow-up are essential for optimal clinical outcomes. The absence of photographic documentation of stages 1 and 2, along with the lack of IKBKG mutation detection, presents limitations that could have strengthened diagnostic certainty. Despite these constraints, this case contributes to the growing understanding of IP in male patients and underscores the importance of ongoing clinical observation and research
